# Urine effects on grass and legume nitrogen isotopic composition: Pronounced short-term dynamics of δ^15^N

**DOI:** 10.1371/journal.pone.0210623

**Published:** 2019-01-16

**Authors:** Bettina Tonn, Ina Porath, Fernando A. Lattanzi, Johannes Isselstein

**Affiliations:** 1 Department of Crop Sciences, Grassland Science, University of Goettingen, Goettingen, Germany; 2 Lehrstuhl für Grünlandlehre, Technische Universität München, Freising-Weihenstephan, Germany; 3 INIA La Estanzuela, Instituto Nacional de Investigación Agropecuaria (INIA Uruguay), Colonia, Uruguay; 4 Centre for Biodiversity and Sustainable Land Use, Goettingen, Goettingen, Germany; Chinese Academy of Sciences, CHINA

## Abstract

Nitrogen stable isotope (^15^N) natural abundance is widely used to study nitrogen cycling. In grazed ecosystems, urine patches are hot-spots of nitrogen inputs, losses, and changes in δ^15^N. Understanding δ^15^N dynamics in urine-affected vegetation is therefore crucial for accurate inferences from ^15^N natural abundance in grasslands. We hypothesized that leaf δ^15^N following urine deposition varies with time and plant functional group. Specifically, we expected (i) short-term decreases in δ^15^N due to foliar absorption of ^15^N-depleted volatilized ammonia, (ii) followed by increases in δ^15^N due to uptake of ^15^N-enriched soil inorganic nitrogen, and (iii) that the magnitude of these changes is less in legumes than in grasses. The latter should be expected because ammonia absorption depends on leaf nitrogen concentration, which is higher in legumes than grasses, and because biological nitrogen fixation will modify the influence of urine-derived nitrogen on δ^15^N in legumes. We applied cattle urine to a mixture of *Lolium perenne* and *Trifolium repens* in a pot experiment. Nitrogen concentration and δ^15^N were determined for successive leaf cohorts and bulk biomass either 17 (early) or 32 (late) days after urine application. Early after urine application, leaves of *L*. *perenne* were ^15^N-depleted compared to control plants (δ^15^N 0.1 vs. 5.8‰, respectively), but leaves of *T*. *repens* were not (-1.1 vs. -1.1‰, respectively). Later, both species increased their δ^15^N, but *T*. *repens* (4.5‰) less so than *L*. *perenne* (5.9‰). Vegetation sampled within and outside urine patches in the field further supported these results. Our findings confirm that foliar ammonia uptake can substantially decrease grass foliar δ^15^N, and that in both grass and legume the direction of the δ^15^N response to urine changes over time. Temporal dynamics of plant δ^15^N at urine patches therefore need to be explicitly addressed when ^15^N natural abundance is used to study nitrogen cycling in grazed grasslands.

## Introduction

The effect of grazing animals on nitrogen cycling is a topic of interest both in natural and agricultural ecosystems. One approach to studying this complex process involves using the natural abundance of the rare stable nitrogen isotope ^15^N as an integrator of the nitrogen cycle [1]. Advances in recent decades have improved our understanding of the link between the isotopic composition of soil, plant and animal nitrogen and the underlying ecological processes [1, 2, 3]. For instance, ecosystem enrichment in ^15^N has been interpreted as the consequence of higher nitrogen loss rates [4, 5], or as an indicator for higher rates of nitrogen cycling [6, 7], because pathways of nitrogen loss discriminate strongly against ^15^N, particularly gaseous losses through ammonia volatilization and denitrification.

In grazed grasslands, animal excreta play a central role in nitrogen turnover [8, 9] creating hot-spots with high rates of gaseous nitrogen losses and nitrate leaching [10–13]. Thus, grazing has been associated with ^15^N enrichment of plants and soil [6, 7, 14]. Close positive relationships (*r^2^* of 0.55–0.86) between ^15^N abundance in soils, plants or animal tissue and stocking rates have been reported in some managed grasslands [4, 15, 16], but not in others [17–20]. This shows how the complexity of the nitrogen cycle in these systems can prevent straightforward, simple inferences between nitrogen cycling and ^15^N abundance.

A little understood aspect of animal excreta is their effect on the temporal δ^15^N dynamics of grasslands. The strong fractionation of ammonia volatilization (ε ~ 40 to 60‰, [1]) results in the emission of highly ^15^N-depleted gaseous ammonia and the retention of highly enriched NH_4_^+^ in the soil solution [1, 3, 13, 21]. The ability of plants to take up gaseous ammonia from the atmosphere by diffusion through stomata [22] has the potential to decrease foliar ^15^N abundance at urine patches. ^15^N depletion through assimilation of gaseous ammonia derived from animal excreta has indeed been reported in other systems [21, 23]. In managed temperate grasslands, up to ~ 25% of the grazing area is affected by urine in a given year [24]. Therefore, if considerable ^15^N depletion regularly occurs at urine patches in grazed grasslands, this may have important implications for the interpretation of ^15^N natural abundance patterns in these systems: hotspots of high nutrient losses might, at least temporarily, be associated with ^15^N depletion rather than enrichment. To gauge the relevance of this process for ^15^N natural abundance studies in grazed grasslands, it is necessary to know over which time period the urine-induced plant biomass depletion in ^15^N may persist.

Further, gaseous ammonia uptake may differ between grasses and legumes, as ammonia uptake is controlled by the ammonia concentration gradient between ambient and substomatal air. The latter is affected by nitrogen nutrition status [25, 26, 27], which is frequently higher in legumes than in grasses [28]. An initial ammonia-induced ^15^N-depletion would be followed by increases in ^15^N abundance caused by uptake of ^15^N-enriched soil nitrogen. Again, this may differ between grasses and legumes because legumes can access atmospheric nitrogen and are thus less dependent on soil-derived nitrogen [29].

**Fig 1:**
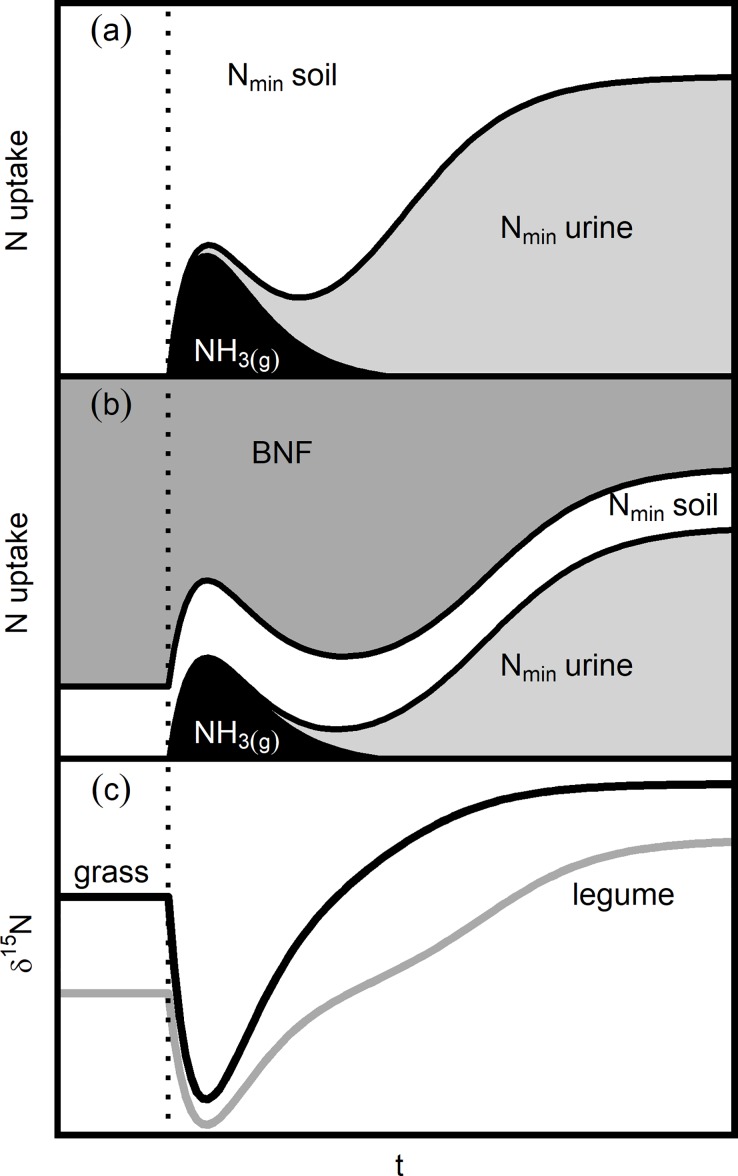
Conceptual graph of hypothesized urine deposition effects on nitrogen uptake and isotopic composition of grass and legume species. Assumed contribution of different nitrogen sources to the total plant nitrogen uptake of (a) a grass, (b) a legume, following urine deposition (vertical dotted line) over time. N_min_ soil: mineral nitrogen from soil nitrogen pool, N_min_ urine: mineral nitrogen derived from urine, NH_3(g)_: nitrogen taken up as gaseous ammonia, BNF: nitrogen from biological nitrogen fixation. The predicted changes of isotopic composition of currently assimilated nitrogen in both grass and legume are shown in (c). The time covered by the figure is approximately three weeks. For more details on the underlying assumptions, see S1 File.

The aim of this study was to understand the short-term dynamics of ^15^N abundance in grasses and legumes affected by urine deposition. Our hypotheses were (i) that plant ^15^N abundance would decrease shortly following urine application because the plants take up ^15^N-depleted gaseous ammonia. We expected (ii) that plant ^15^N abundance would subsequently increase, reflecting the uptake of enriched inorganic nitrogen from the soil solution. Finally, we expected (iii) that variation in ^15^N abundance would be lesser in the nitrogen-fixing legume *Trifolium repens* L. than in the grass *Lolium perenne* L. These hypotheses are conceptually summarized in Fig 1.

## Materials and methods

### Pot experiment

The experiment was conducted in a greenhouse without artificial lighting. The two experimental factors, urine application (urine applied / untreated control) and harvest date (early / late), were assessed in a completely randomized design with four replications (pots). The greenhouse had one open and three closed sides. Pots were placed on tables that could be moved outside the greenhouse when stable weather conditions allowed.

The species used were the grass *Lolium perenne* cv. ‘Signum’ and the legume *Trifolium repens* cv. ‘Rivendel’. Seven to ten days after sowing, from 17 to 20-Apr-2012, fifteen seedlings of *L*. *perenne* and ten seedlings of *T*. *repens* each were planted together into square pots measuring 18x18x18 cm. A nitrogen-poor growing substrate was produced by mixing potting soil (sandy loam, pH (CaCl_2_) 6.8, 0.45 g kg^-1^ extractable phosphorus (calcium acetate lactate), 1.13 g kg^-1^ extractable potassium (CaCl_2_), 97 g kg^-1^ organic matter, 62 g kg^-1^ carbon, 4.5 g kg^-1^ total nitrogen, δ^15^N of total nitrogen 5.1‰) with sand in the ratio of 1:6 (weight-based). To ensure uniform establishment of seedlings, the top 1.5 cm layer consisted entirely of potting soil. Before the experiment started, plants were cut to a height of 5 cm twice, 27 and 45 days after sowing.

Three weeks later, on 15-Jun-2012, cattle urine was applied to half of the pots. Before urine application, pots of the urine treatments were separated from those of the control treatment by a distance of at least 10 m, to avoid uptake of gaseous ammonia by plants in the control treatment. Per pot, 300 ml of cattle urine was then added in the urine treatment, while the control received an equal amount of distilled water. Both liquids were applied close to the soil surface using a wash bottle and were spread uniformly over the pot surface without wetting aboveground plant parts. After four days, pots of the two treatments were joined again.

The urine that was applied was collected from five Simmental cows of the university-owned stocks at the Research Farm Relliehausen (University of Goettingen), located at the Solling uplands (51° 47’ N, 9° 42’ E). Care and housing conditions of the cattle were in line with German regulations: animal welfare act (TierSchG) and Tierschutz-Nutztierhaltungsverordnung (TierSchNutztV). The University’s Animal Welfare and Ethics Committee approved the use of urine samples from cattle in this study (approval no. E1-18). The animals’ diet during the weeks preceding the sampling partly consisted of grazed pasture and partly of grass silage. To collect urine, the animals were temporarily kept in place using the headlock of the feed fence of their barn, with regular access to feed and water. When animals voluntarily started to urinate, urine was captured with a bucket. It was then immediately stored at cool temperatures and frozen before being applied. At application, a subsample was taken for analysis. An amount of 180 ml taken from the urine subsample was acidified by adding nitric acid, freeze dried and analyzed for its nitrogen concentration and isotopic composition (see below). δ^15^N of the urine applied was determined as 2.0‰, while nitrogen concentration was 5.6 g l^-1^. The nitrogen application rate in the urine treatment was therefore 1.68 g per pot, corresponding to 52 g m^-2^, which can be considered as typical for naturally occurring urine patches [9].

Two pots were set up for each treatment. One was used to harvest total aboveground biomass either 17 days (early harvest) or 32 days (late harvest) after the urine application (2-Jul-2012 and 17-Jul-2012, respectively). Plants were cut at soil surface level and sorted into *L*. *perenne* and *T*. *repens*. The second pot was used to monitor and harvest successive leaf cohorts, *i*.*e*. leaves of comparable developmental stage. For this, five tillers of *L*. *perenne* and five leaves of *T*. *repens* were marked using coloured wire on 12 to 13-Jun-2012. Tillers of *L*. *perenne* were chosen so that the lamina of the youngest growing leaf had a visible length of 2–4 cm. The next older leaf was permanently marked with a tip of correcting liquid. The leaves marked in this way made up the first leaf cohort of *L*. *perenne* that was sampled later. Leaves of *T*. *repens* were marked on stolons whose youngest leaf was in the developmental stage 0.6 on the zero-to-one scale according to Carlson [30]. The next older leaf of that stolon was marked with wire and was part of the first leaf cohort of *T*. *repens*. During the course of the experiment marked tillers and stolons were monitored in intervals of three to seven days. The visible lamina length of marked *L*. *perenne* leaves and subsequently developing leaves of the same tiller was measured. Developmental stage according to Carlson [30] of marked *T*. *repens* leaves and leaves subsequently developing on the same stolon were recorded. Leaves of newer cohorts were marked as older leaves became senescent. Using these measurements, the main period of growth for the leaf cohorts harvested during the experiment could be estimated (S1 Fig). At the early harvest, laminae of the first three leaf cohorts of *L*. *perenne* were sampled from all marked tillers. Additionally, entire leaves of the third leaf cohort of marked stolons of *T*. *repens* were collected. At the second harvest date, laminae of leaf cohorts three and four were collected from *L*. *perenne* as well as *T*. *repens* leaves from the fifth leaf cohort. All harvested samples were dried for 48 h at 60°C, and their dry mass was determined. Both *L*. *perenne* and *T*. *repens* remained in a vegetative state throughout the experiment.

A temperature sensor was suspended 40 cm above the soil surface of the experimental plots and temperature was logged in 15-min intervals. During the period of 48 h following urine application, average temperature was 21.3°C, with maximum temperatures of 26.6°C within the first 24 h after urine application, and 39.1°C within the following 24 h. Average temperature was 21.5°C between urine application and first harvest, and 21.3°C between first and second harvest.

### Field sampling

Field samples were taken at the Grünschweige Grassland Research Station (48° 23′ N, 11° 50′ E, 435 m above sea level) near Freising, Germany, on 23-Aug-2010. The sampled paddock was part of a long-term grazing experiment and was kept at a target sward height of 5 cm through continuous cattle grazing. For details on experimental site and grazing management see [31]. The δ^15^N of topsoil total nitrogen was 4.5 ‰. For this, in spring several soil cores (5 cm internal diameter, 10 cm depth) were taken from the paddock. All aboveground plant biomass was clipped and the soil cores were pooled. Soil samples were sieved through a 2 mm sieve, separating all visible roots, and then air-dried. Dried soil was analysed for δ^15^N as described below. At the time of sampling, recent urine patches could be distinguished by a distinctly darker green colour and higher sward height. Six urine patches and additionally two control patches without visible influence of animal excrements were selected. In each of the eight patches, ten vegetative tillers of *L*. *perenne* were chosen randomly. The laminae of the youngest fully developed leaf and the most recent dead leaf of these tillers were collected and immediately dried at 60°C for 24 hours. *T*. *repens* had low cover in fresh urine patches, and few dead leaves could be found. When available, youngest dead leaf and first fully extended leaf of ten stolons were sampled in the same patches as *L*. *perenne*, and treated in the same way. *L*. *perenne* was in a vegetative state at the time of sampling, while *T*. *repens* was flowering.

### Sample preparation and analysis

All plant samples were dried to constant weight at a temperature of 60°C in a forced-air oven. Soil samples were dried at room temperature over several days. All samples were ground using a ball mill. Samples from the pot experiment were analyzed for their nitrogen concentration and nitrogen isotope abundances using an isotope ratio mass spectrometer (Delta Plus, Finnigan MAT), linked with a Conflo III-Interface (Finnigan MAT, Bremen, Germany) to an elemental analyzer (Carlo Erba NA 1110, Milan, Italy), with Acetanilid as an internal standard. Analysis of the samples from the field experiment followed the same procedure, except that a Conflo II-Interface (Finnigan MAT, Bremen, Germany) was used and that the internal standard was flour. In both cases, long-term precision for δ^15^N of the internal lab standards was better than ± 0.2‰. Nitrogen isotope abundances are presented using δ notation [δ^15^N (‰) = (R_sample_/R_standard_)– 1, where R is the isotope ratio ^15^N/^14^N, and the standard is atmospheric N_2_].

### Statistical analysis

The urine application response of leaf nitrogen concentration and δ^15^N of the different leaf cohorts in the pot experiment was analyzed using linear mixed effects models. Fixed effects were leaf cohort, urine application, and their interaction. Experimental pot was treated as a random effect. The results from the two leaf sampling dates were analyzed separately. The factor leaf cohort had four levels at the first sampling date (first, second and third leaf cohort of *L*. *perenne*, third leaf cohort of *T*. *repens*) and three levels at the second harvest date (third and fourth leaf cohort of *L*. *perenne*, fifth leaf cohort of *T*. *repens*).

Two separate types of analysis were performed for the total aboveground biomass harvest of the pot experiment. Firstly, linear mixed effects models were performed with the single and interactive effects of urine application, harvest date and species as fixed effects and pot as a random effect in order to assess the response of biomass nitrogen concentration and δ^15^N. Secondly, values for total harvested biomass, species-weighted mean nitrogen concentration and δ^15^N as well as yield percentage of *T*. *repens* were calculated for each pot. The response of these parameters to urine application, harvest date and their interaction was tested using generalized least squares models.

Analyses were performed with the statistical software R 3.2.3 [32] and the package “nlme” [33]. In all cases, residuals were inspected for homoscedascity and normality. Where necessary, variance structure was adapted as indicated in footnotes to Tables 1, 2 and 3. Minimum adequate models were selected by sequentially removing fixed effects to reach a minimum value of the Second-order Akaike Information (AICc) as calculated by the package “MuMIn” [34]. Significance of the fixed effects remaining in the model was tested using sequential Wald tests. For significant predictors, post-hoc tests were performed as Tukey tests using the package “lsmeans” [35]. A significance level of *P* < 0.05 was chosen throughout.

**Table 1:** Effects of urine application and harvest date on yield, botanical and chemical composition of the *Trifolium-repens-Lolium-perenne* mixture.

Harvest	Urine	Dry matter yield (g)	N yield (mg)*	Proportion of *T*. repens*	N (%)*	δ^15^N (‰)*
early	no	13.0 ^c^	316 ^c^	0.36	2.42	4.0 ^a^
	yes	12.4 ^c^	501 ^bc^	0.15	3.93	1.1 ^b^
late	no	20.0 ^b^	505 ^b^	0.51	2.54	2.4 ^b^
	yes	25.9 ^a^	944 ^a^	0.19	3.65	5.1 ^a^
urine		*F*_1,12_ = 4.12 *p* = 0.065	*F*_1,12_ = 30.97 *p* < 0.001	*F*_1,13_ = 37.70 *p* < 0.001	*F*_1,14_ = 185.28 *p* < 0.001	*F*_1,12_ = 0.03 *p* = 0.875
harvest		*F*_1,12_ = 59.90 *p* < 0.001	*F*_1,12_ = 46.42 *p* < 0.001	*F*_1,13_ = 4.84 *p* = 0.047	-	*F*_1,12_ = 72.73 *p* < 0.001
urine × harvest		*F*_1,12_ = 5.95 *p* = 0.031	*F*_1,12_ = 5.12 *p* = 0.043	-	-	*F*_1,12_ = 42.94 *p* < 0.001

Aboveground dry matter yield and nitrogen yield per pot, biomass proportion of *Trifolium repens* and biomass nitrogen concentration and isotopic composition of mixed cultures of *T*. *repens* and *Lolium perenne*, as influenced by harvest date and urine application. Results of generalized least squares models (*F* values, *p* values). Where the urine × harvest interaction is significant, different letters designate significant differences between means.

* The variance structure varIdent was used in the model (allowing for differing variances for each urine treatment).

**Table 2:** Effects of urine application and leaf cohort on nitrogen concentration and nitrogen isotopic composition of *Lolium perenne* and *Trifolium repens* leaves.

Effect	df	N		δ^15^N	
		*F*	*p*	*F*	*p*
early harvest ^a^					
Urine	1,6	81.81	< 0.001	63.30	< 0.001
Leaf	3,18	14.34	< 0.001	257.43	< 0.001
urine × leaf	3,18	23.18	< 0.001	5.66	0.007
late harvest ^b^					
urine	1,6	232.19	< 0.001	311.37	< 0.001
leaf	2,12	86.01	< 0.001	223.45	< 0.001
urine × leaf	2,12	6.29	0.014	12.21	0.001

*F* and *p* values of the linear mixed effects models testing the effects of urine application and leaf cohort on leaf nitrogen concentration and leaf nitrogen isotopic composition at two different harvest dates.

^a,b^ The variance structure varIdent was used in the models, allowing for differing variances for each urine treatment (^a^) or each combination of urine treatment and species (^b^).

**Table 3:** Effects of urine application on nitrogen concentration and nitrogen isotopic composition of *Lolium perenne* and *Trifolium repens* aboveground bulk biomass.

Effect	df	*F*	*p*
N concentration			
urine	1,13	118.25	< 0.001
species	1,14	282.71	< 0.001
harvest	1,13	3.55	0.082
urine × species	1,14	137.10	< 0.001
δ^15^N			
urine	1,12	0.07	0.802
species	1,14	5971.21	< 0.001
harvest	1,12	9.91	0.008
urine × species	1,14	73.36	< 0.001
urine × harvest	1,12	56.82	< 0.001

*F* and *p* values of the linear mixed effects models testing the response of aboveground biomass nitrogen concentration and isotopic composition of *Lolium perenne* and *Trifolium repens* to urine application, depending on harvest date. The variance structure varIdent was used in both models, allowing for differing variances for each urine treatment.

## Results

### Apparent nitrogen recovery and productivity response to urine application

An increased aboveground dry matter yield in response to urine application was only observed at the late harvest (Table 1). Aboveground nitrogen yield showed a non-significant numerical increase by 58% at the early harvest, and a significant increase by 87% at the late harvest in the urine compared to the control treatment (Table 1). Apparent recovery of urine nitrogen was calculated as the difference between nitrogen yield with and without urine application, divided by amount of nitrogen applied via urine. It equalled 0.11 g g^-1^ at the early and 0.26 g g^-1^ at the late harvest date. The biomass proportion of *T*. *repens* was decreased by urine application, yet increased from the early to the late harvest date (Table 1).

### Nitrogen concentration and isotopic composition of leaf cohort harvests

**Fig 2:**
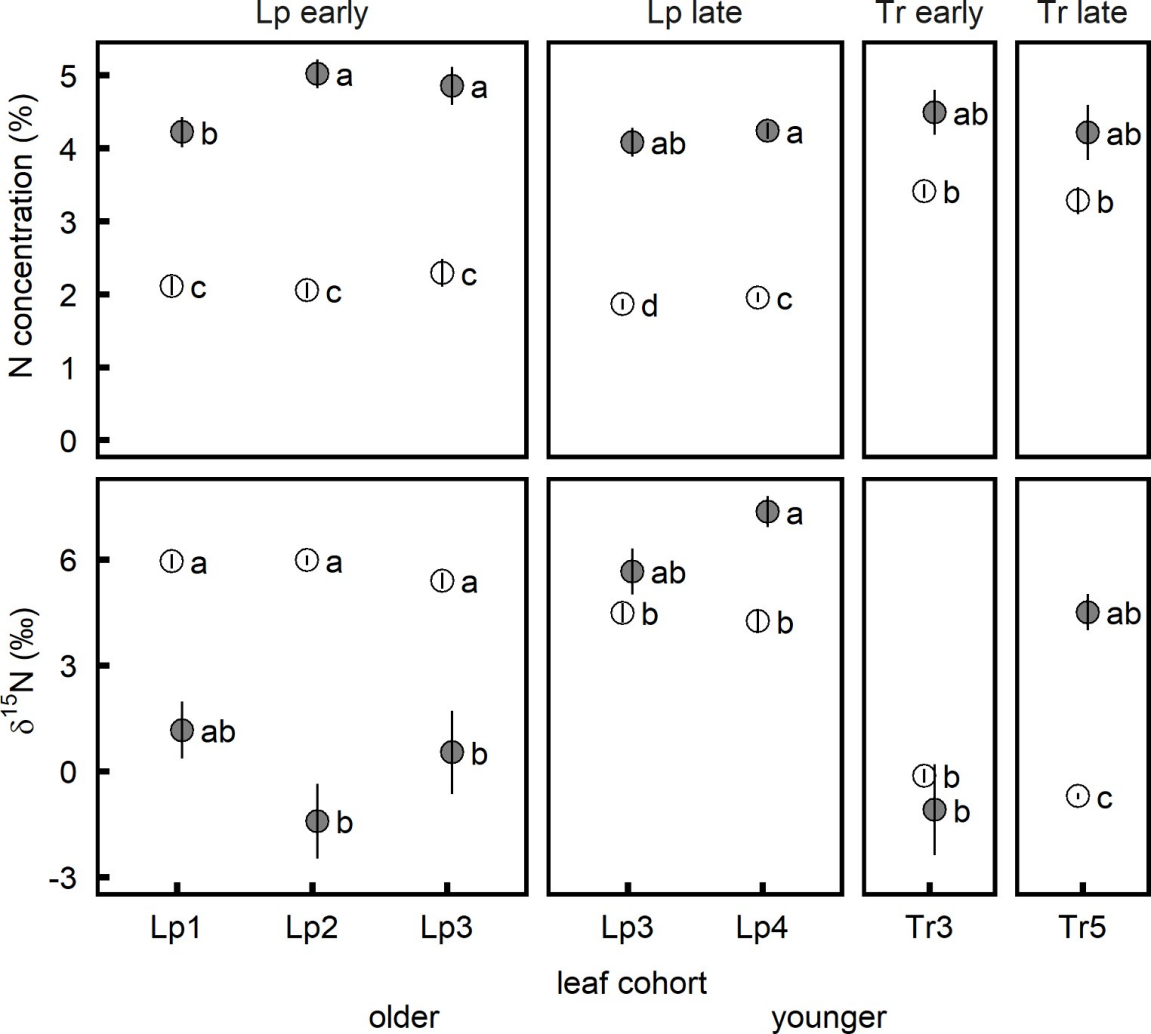
Nitrogen concentration and nitrogen isotope composition of *Lolium perenne* and *Trifolium repens* leaves as affected by urine application. Laminae of four successive leaf cohorts of *L*. *perenne* (Lp1, Lp2, Lp3, Lp4) and leaves of two leaf cohorts of *T*. *repens* (Tr3, Tr5) were harvested at two dates. Grey: urine, white: control; error bars: standard error of the mean. Different letters mark significant (*p* < 0.05) differences between mean values within each harvest date.

At both harvest dates, the urine treatment response of leaf nitrogen concentration differed between leaf cohorts (Table 2). Urine application increased the nitrogen concentration in all leaf cohorts of *L*. *perenne*, but in neither of the two harvested leaf cohorts of *T*. *repens* (Fig 2). Nitrogen concentrations were lower in *L*. *perenne* than in *T*. *repens* leaves in the control treatment (Fig 2). In the urine treatment, however, *L*. *perenne* leaves generally had the same nitrogen concentrations as the simultaneously harvested leaves of *T*. *repens* (Fig 2).

Like nitrogen concentration, δ^15^N also consistently varied between leaves of *L*. *perenne* and *T*. *repens* in the control treatment. With an average value of 5.8 and 4.4‰, *L*. *perenne* leaves were more enriched in ^15^N than leaves of *T*. *repens* with values of -0.1 and -0.7‰ in the early and late harvests, respectively (Fig 2). Again, there was a significant interaction between leaf cohort and urine treatment (Table 2). The second and third leaf cohort of *L*. *perenne*, collected at the early harvest date, had a significantly lower δ^15^N than the equivalent leaves of the control (Fig 2). On average, δ^15^N of urine-treated *L*. *perenne* leaves harvested at that date was as low as 0.1‰. The isotope composition of *T*. *repens* leaves was not affected by urine application at the first harvest (Fig 2). A different response was observed in the leaves collected at the second harvest, two weeks later. Urine application lead to an increased δ^15^N in the fourth leaf cohort of *L*. *perenne* and in the sampled *T*. *repens* leaves, and no longer lead to a difference between urine and control treatment in the third leaf cohort of *L*. *perenne* (Fig 2).

### Nitrogen concentration and isotopic composition of aboveground bulk biomass of *L*. *perenne* and *T*. *repens*

**Fig 3:**
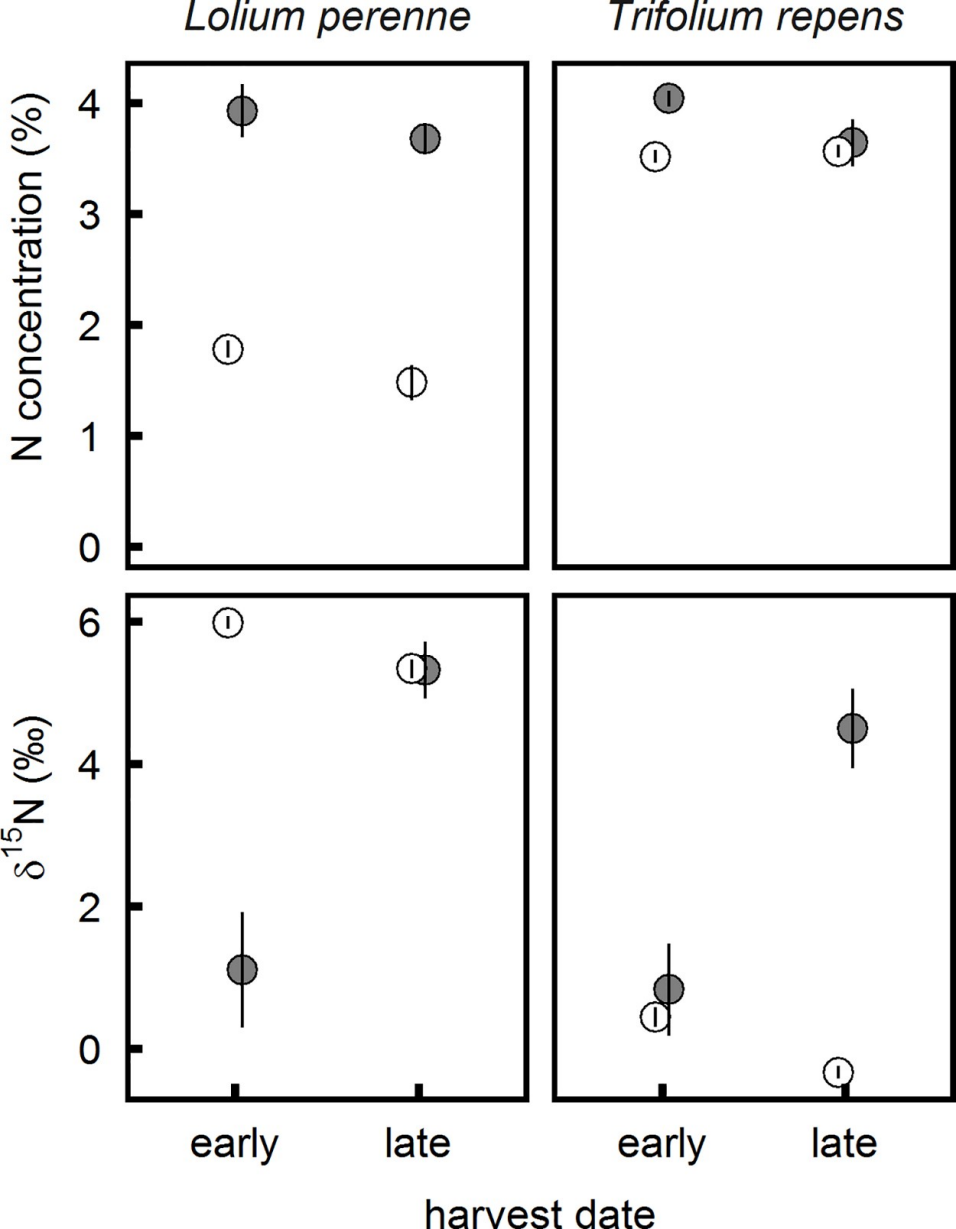
Nitrogen concentration and nitrogen isotope composition of *Lolium perenne* and *Trifolium repens* bulk biomass as affected by urine application and harvest date. Total aboveground biomass of the *Lolium-perenne-Trifolium-repens* mixture. Grey: urine, white: control; error bars: standard error of the mean.

Results obtained from the bulk biomass harvests were qualitatively similar to those of the leaf cohort harvests (Fig 3, S2 Fig). In the control treatment, nitrogen concentrations of bulk biomass were always lower in *L*. *perenne* than in *T*. *repens* (Fig 3). Urine application raised nitrogen concentrations in *L*. *perenne* to the same level as those of *T*. *repens*, which were unaffected by urine (Fig 3). This resulted in an increased nitrogen concentration in total aboveground biomass in the urine treatment, with no effect of harvest date (Table 1).

For δ^15^N, the interactions of urine treatment with species and with harvest date were both significant (Table 3). Averaged over harvest dates, δ^15^N means of the urine treatment × species combinations increased significantly in the order of Control *T*. *repens* < Urine *T*. *repens* = Urine *L*. *perenne* < Control *L*. *perenne*. Averaged over species, δ^15^N means of urine treatments increased significantly in the order of Urine Early < Control Late < Urine Late < Control Early. As a result of these two interactions, the urine treatment lead to a much lower δ^15^N of *L*. *perenne* at the first harvest, compared to the corresponding control treatment or to both treatments at the late harvest (Fig 3). Conversely, in the second harvest, *T*. *repens* in the urine treatment was enriched in ^15^N compared to the control treatment or to both treatments at the earlier harvest (Fig 3). These species-specific reactions combined with the response of the species’ relative contribution to aboveground biomass resulted in a significant interaction between urine treatment and harvest date on the level of total biomass δ^15^N (Table 1). The lowest mean δ^15^N of 1.1 and 2.4‰, respectively, were observed in the urine treatment at the early and in the control treatment at the late harvest (Table 1). These values were significantly lower than the δ^15^N of the control treatment at the early and the urine treatment at the late harvest (4.0 and 5.1‰ respectively, Table 1). Both nitrogen concentrations and isotopic composition varied more strongly between replications in the urine than in the control treatment (Fig 3).

### Nitrogen concentration and isotopic composition of *L*. *perenne* and *T*. *repens* in the field

**Fig 4:**
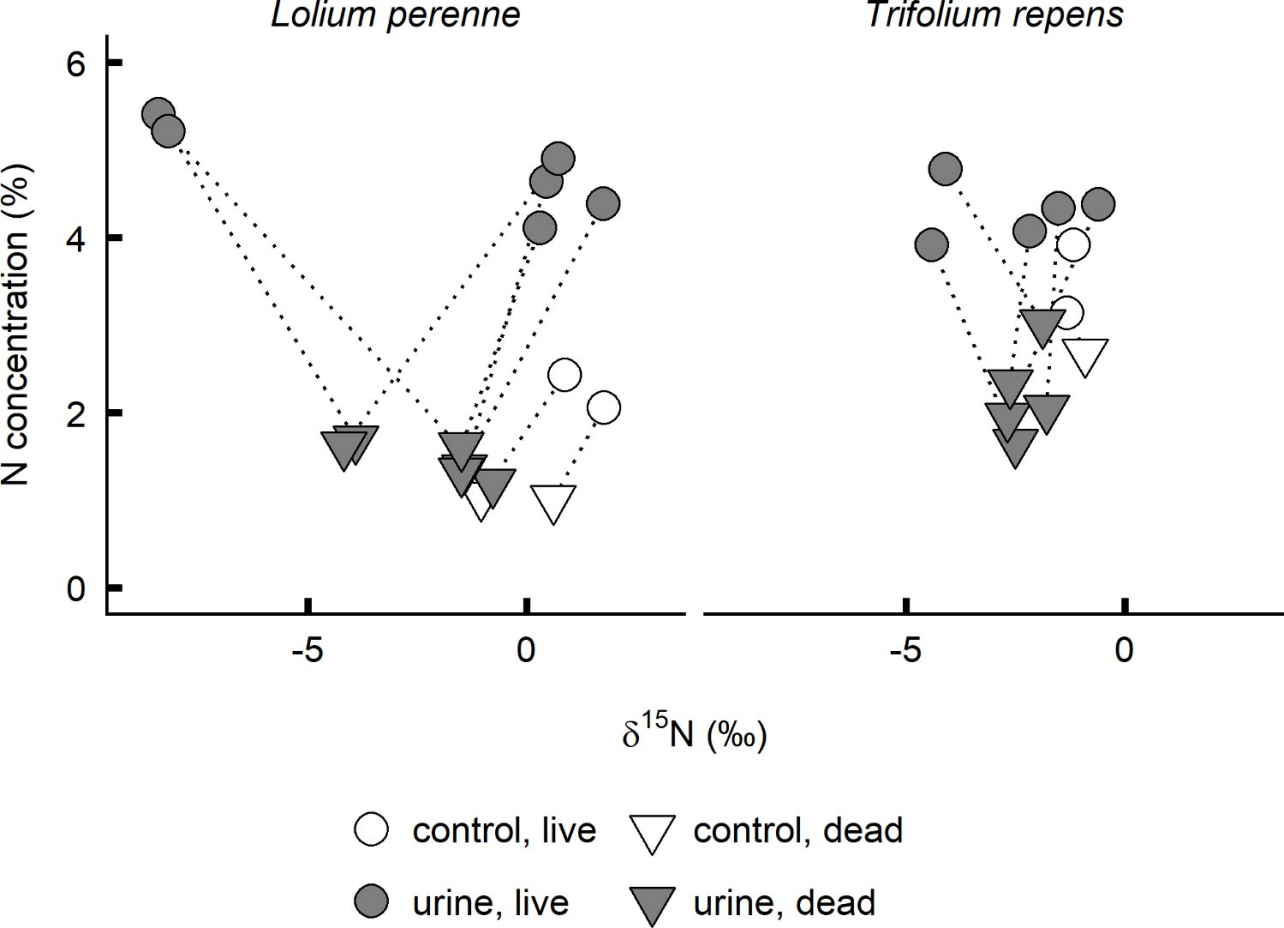
Nitrogen concentration and isotopic composition of *Lolium perenne* and *Trifolium repens* leaves sampled on a pasture. Live and dead laminae of *L*. *perenne* or leaves of *T*. *repens* were sampled at six urine patches (five in case of *T*. *repens*) and two control patches not recently affected by urine. Values of live and dead leaves collected at the same patch are connected with a dotted line.

Nitrogen concentration was generally higher in live *L*. *perenne* leaves sampled at urine (4.78 ± 0.48%, mean ± standard deviation) compared to control patches (2.25 ± 0.27%, Fig 4). While δ^15^N of live *L*. *perenne* leaves ranged between 0.3 and 1.8‰ in control patches and the majority of urine patches, at two urine patches strongly negative values of -8.2 and -8.4‰ were found (Fig 4). Dead *L*. *perenne* leaves had lower nitrogen concentrations and tended to be slightly depleted compared to the corresponding live leaves (Fig 4). Again, at two urine patches δ^15^N was conspicuously less (-3.9 and -4.2‰) than the range observed at the remaining patches (-1.5 to 0.6‰, Fig 4). Leaves of *T*. *repens* showed a lower variability both in terms of nitrogen concentration and δ^15^N (Fig 4).

## Discussion

### δ^15^N dynamics in a grass and a nitrogen-fixing legume following urine application

Urine deposition more than doubled nitrogen concentration in bulk biomass of *L*. *perenne* 17 days after application, and decreased its δ^15^N relative to the untreated control (Fig 2). Fifteen days later, δ^15^N of the bulk biomass increased, returning to control levels (Fig 2). This supports the idea that uptake of volatilized ammonia in urine patches decreases δ^15^N of shoot biomass shortly after urine application, but then nitrogen taken up from the soil becomes more enriched as time after urine deposition passes. This agrees with results from individual leaves: while the initial depletion was similar in all three leaf cohorts, subsequent enrichment was most marked in the youngest leaf cohort sampled, which started to emerge between one and two weeks after urine application (Fig 3, S1 Fig).

Ammonia volatilization strongly fractionates against ^15^N (ε of ~ 40 to 60‰, [1]). As in any Rayleigh process, the actual isotopic composition of volatilized ammonia may vary considerably, depending on the completeness of the reaction. Average δ^15^N values of -36 to -18‰ have been observed in ammonia lost from urine patches [13, 21]. Eriksen and Høgh-Jensen [36] attributed decreases in δ^15^N in shoots of *L*. *perenne* two weeks after the application of artificial urine to either exchange between urine-derived ammonium and depleted adsorbed soil ammonium or to fractionation during urea hydrolysis. Given the high proportion of sand in our substrate, exchange of adsorbed ammonium is unlikely to have played a quantitatively important role in our experiment. Further, as urea hydrolysis would have been relatively fast [37], effects of fractionation would have become negligible since they are equalized once the reaction is completed. Thus, uptake of depleted ammonia best explains the ^15^N depletion we observed in *L*. *perenne* and might also have contributed to the results of Eriksen and Høgh-Jensen [36].

As expected, the dynamics of δ^15^N in *T*. *repens* differed from those in *L*. *perenne*. Firstly, urine deposition had almost no effect on nitrogen concentration (Fig 2, Fig 3) of *T*. *repens*. Secondly, neither bulk aboveground biomass nor individual leaf cohorts of *T*. *repens* showed a δ^15^N response to urine deposition at the first harvest date (Figs 2 and 3). Thirty-two days after urine application, however, δ^15^N did increase markedly in *T*. *repens* (Figs 2 and 3).This indicates that uptake of soil nitrogen had overridden biological nitrogen fixation as a source of nitrogen. Such substantial reductions in the contribution of biological nitrogen fixation to nitrogen uptake of *T*. *repens* have previously been observed to last for two to five months after urine deposition [22, 23].

Although the interpretation of changes in δ^15^N of *T*. *repens* is complicated by the unknown proportion and isotopic composition of the biologically fixed nitrogen [2, 3], the results suggest that uptake of gaseous ammonia in *T*. *repens* was less than in *L*. *perenne*. In our experiment, as in most grasslands, *T*. *repens* had higher nitrogen concentrations than *L*. *perenne* (Fig 3). This would have minimized net stomatal ammonia uptake, because higher nitrogen nutrition status increases ammonia concentration in substomatal air [25, 26, 27]. Additionally, a smaller leaf area and therefore absorption surface, or also lesser stomatal conductivity of *T*. *repens* during the first days after urine application, may have further contributed to species differences in ammonia assimilation [22].

### Relationship between field and greenhouse results, and implications for the use of ^15^N natural abundance in studies of grazed systems

Plant biomass of grazed grasslands is highly variable in nitrogen isotopic composition [36, 38]. A controlled-environment experiment allowed testing hypotheses about short-term variations in δ^15^N of grassland plants in response to urine deposition. Both the microclimate in the greenhouse and the substrate used in the pot study may have potentially affected ammonia emissions and assimilation. Temperatures were higher in the greenhouse than outside, which would have somewhat increased cumulative ammonia volatilization [39]. Absence of wind may have led to lesser air exchange within the plant canopy and therefore to increased ammonia concentrations, favouring assimilation but at the same time reducing cumulative ammonia volatilization [39]. To maximize the importance of urine-derived or biologically fixed nitrogen for the nutrition of our experimental plants, we used a substrate with low organic matter concentration. In soils with higher organic matter concentration, a smaller proportion of ammonia might be volatilized, and higher rates of mineralization might dampen the ^15^N enrichment subsequent to urine deposition [13]. While field conditions therefore might have decreased ammonia assimilation and consequently the size of the early urine addition effect on biomass δ^15^N compared to our results, they would not have changed the pattern of rapid depletion and subsequent enrichment that was observed in *L*. *perenne* (Figs 2 and 3) and in the mixed culture biomass (Table 1).

Our field data support that ammonia assimilation may affect foliar nitrogen isotopic composition following urine deposition. Live leaves of *L*. *perenne* sampled from urine patches had highly variable δ^15^N values, some leaves as low as -8.4‰ (compared to a mean of about 1‰ in non-affected patches; Fig 4). This is very similar to the maximum difference in δ^15^N observed between the most depleted leaves of the urine treatment (-3.1‰) and the mean value of the corresponding control leaves (6.0‰) in the pot experiment (Fig 2). Notably, the most depleted leaves sampled in the field were those with the highest nitrogen concentration. Based on the results of the pot experiment, this suggests more recent urine deposition: at the first harvest of the pot experiment, nitrogen concentration and δ^15^N of *L*. *perenne* leaves in the urine treatment were negatively correlated (Pearson’s *r* = -0.67, *df* = 6, *p* = 0.001), while the correlation tended to be positive (Pearson’s *r* = 0.71, *df* = 6, *p* = 0.051) fifteen days later, at the second harvest. *T*. *repens* leaves in the field showed neither clear depletion nor enrichment in plants growing in urine *vs*. non-urine patches (Fig 4). Experiments sampling urine patches of known age would be helpful to assess magnitude and temporal dynamics of the nitrogen isotopic composition changes that occur after urine deposition in the field in greater detail.

Our study shows that the time-lag following urine deposition as well as plant functional group affect nitrogen isotopic composition. Being aware of the temporal sensitivity of biomass δ^15^N to urine deposition can be important when making inferences about nitrogen cycling in grazed grasslands. For instance, urine deposition disrupted the usual relationship observed between legume biomass proportion and bulk biomass nitrogen concentration (positive) and isotopic composition (negative). This is because urine affects nitrogen concentration and isotopic composition of grasses much more than that of legumes, and because the magnitude of this effect varies with time (S3 Fig). Likewise, a grazing study [18] observed differences in δ^15^N of up to -8‰ between successive leaf cohorts of several grassland species in Inner Mongolia. Given that biomass was sampled in continuously stocked pastures, urine patches might have been responsible for part of that variability.

Consequently, in addition to the non-trivial problem of representatively sampling urine and non-urine patches [24, 40], time lag between probable urine deposition and sampling date has to be considered as well in grazing studies. The observed temporal dynamics of nitrogen isotopic composition after urine deposition might, however, also be utilized as temporal tracers of urine deposition events. Results from our pot experiment (Fig 2) indicate that nitrogen isotopic composition of consecutive leaf cohorts, linked to leaf appearance rates, might be used to retrospectively determine time lag to urine deposition in visually determined recent urine patches.

## Conclusions

In line with our initial expectation, urine application led to strong temporal dynamics in biomass nitrogen isotope composition, with different responses in the investigated grass and legume species. Depending on the time lag between urine deposition and biomass sampling, plant δ^15^N at urine patches may show very different responses, with the short-term response diametrically opposed to assumptions generally made in nitrogen cycling studies of grazed grasslands. The generally expected ^15^N enrichment was only observed one month after urine application, most clearly in the legume. Two weeks earlier, the grass species was found to be strongly depleted in ^15^N, and isotopic composition of the legume to be unaffected by urine addition. We therefore conclude that not only spatial, but temporal heterogeneity in nitrogen cycling caused by urine patches needs to be explicitly addressed when using ^15^N natural abundance in grazing studies.

## Supporting information

S1 Fig Development of leaf length of successive leaf cohorts of *Lolium perenne* over time. (PDF)

S2 Fig Relationship between leaf and bulk aboveground biomass values of nitrogen concentration and isotopic composition. (PDF)

S3 Fig Relationship between the proportion nitrogen contained in *Trifolium repens* and mixture nitrogen concentration / isotope composition. (PDF)

S1 File Assumptions and R code underlying Fig 1. (TXT)

S2 File Original data pot experiment–leaf cohorts. (CSV)

S3 File Original data pot experiment–bulk biomass. (CSV)

S4 File Original data field observations. (CSV)
