# Supplementary material for: Urine effects on grass and legume nitrogen isotopic composition: Pronounced short-term dynamics of δ15N
Source: PLoS One. 2019 Jan 16;14(1):e0210623. doi: 10.1371/journal.pone.0210623 (PMC6334936; doi:10.1371/journal.pone.0210623)
Supplement: S1 Fig — (PDF) [file pone.0210623.s001.pdf]

# S1 Figure

**Urine patches induce species-specific short-term $^{15}N$ depletion of aboveground biomass – consequences for the interpretation of $^{15}N$ signature in nutrient cycling studies of grazing systems**

Bettina Tonn, Ina Porath, Fernando A. Lattanzi, Johannes Isselstein

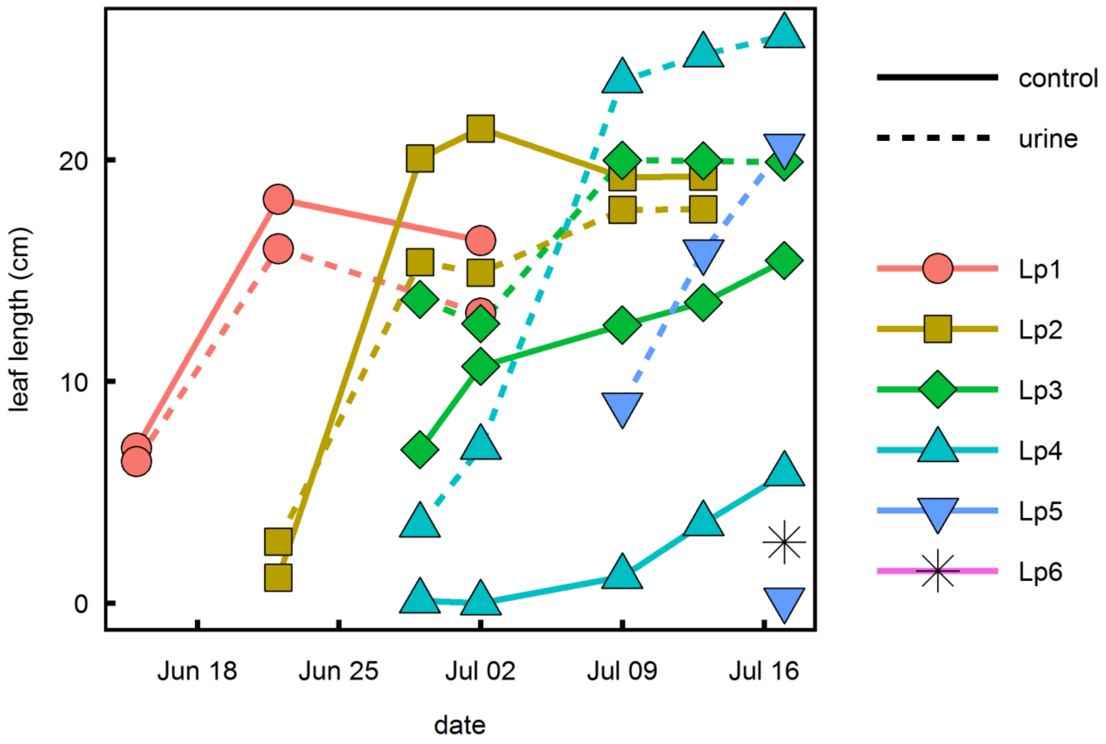


**S1 Fig. Development of leaf length of successive leaf cohorts of *Lolium perenne* over time.** Length measured from the collar of the subtending leaf.
