# Supplementary material for: Urine effects on grass and legume nitrogen isotopic composition: Pronounced short-term dynamics of δ15N
Source: PLoS One. 2019 Jan 16;14(1):e0210623. doi: 10.1371/journal.pone.0210623 (PMC6334936; doi:10.1371/journal.pone.0210623)
Supplement: S2 Fig — (PDF) [file pone.0210623.s002.pdf]

S2 Figure

Urine patches induce species-specific short-term ^{15}N depletion of aboveground biomass – consequences for the interpretation of ^{15}N signature in nutrient cycling studies of grazing systems

Bettina Tonn, Ina Porath, Fernando A. Lattanzi, Johannes Isselstein

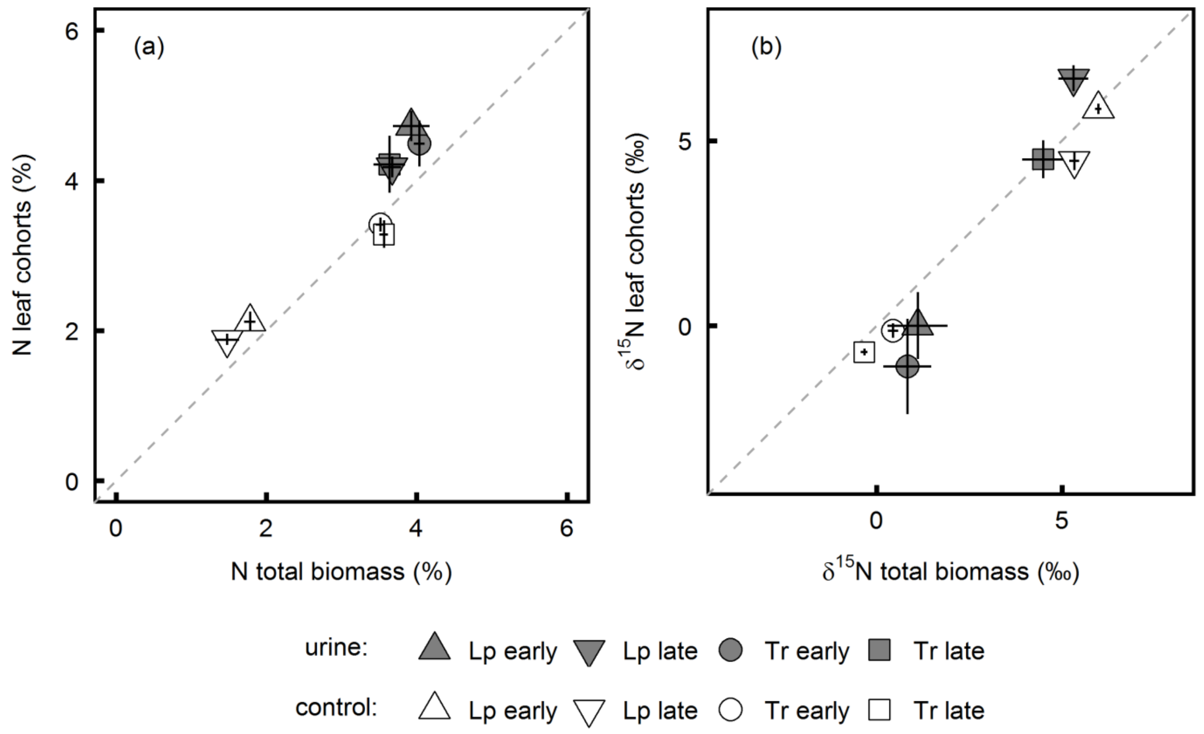

S2 Fig. Relationship between leaf and bulk biomass values of nitrogen concentration and isotopic composition.

Relationship between nitrogen concentration (a) / isotopic composition (b) as a weighted mean of values in harvested leaf cohorts and bulk aboveground biomass of *L. perenne* and *T. repens* at early and late harvest depending on urine application treatment; error bars: standard error of the mean.
