# Supplementary material for: Urine effects on grass and legume nitrogen isotopic composition: Pronounced short-term dynamics of δ15N
Source: PLoS One. 2019 Jan 16;14(1):e0210623. doi: 10.1371/journal.pone.0210623 (PMC6334936; doi:10.1371/journal.pone.0210623)
Supplement: S3 Fig — (PDF) [file pone.0210623.s003.pdf]

S3 Figure

Urine patches induce species-specific short-term ^{15}N depletion of aboveground biomass – consequences for the interpretation of ^{15}N signature in nutrient cycling studies of grazing systems

Bettina Tonn, Ina Porath, Fernando A. Lattanzi, Johannes Isselstein

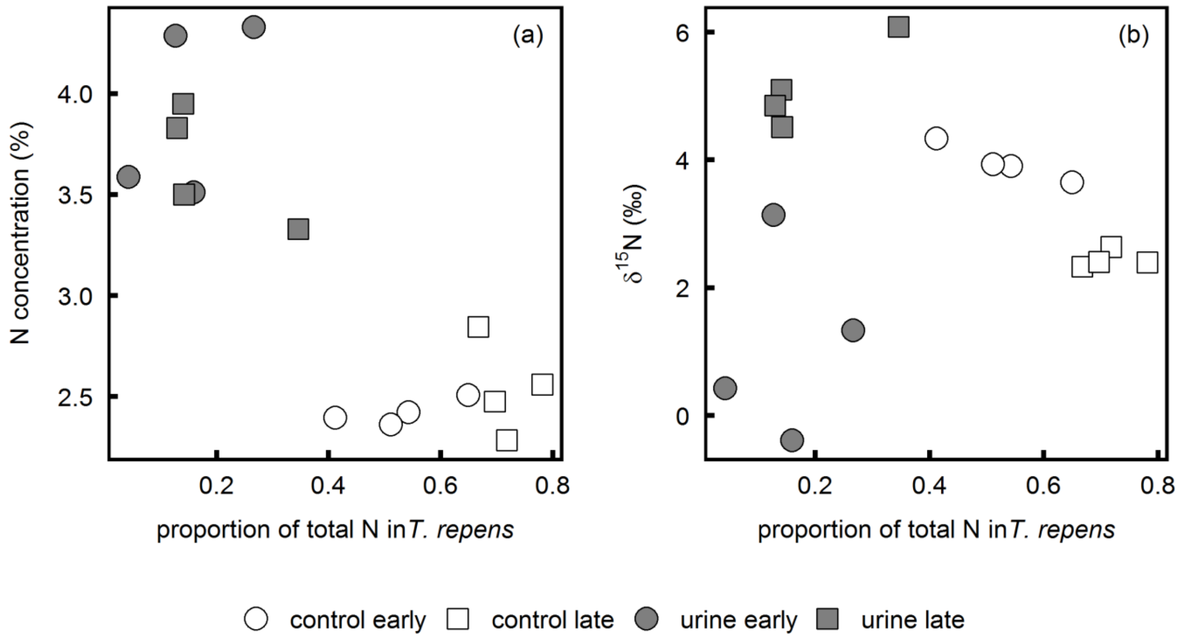

S3 Fig. Relationship between the proportion of nitrogen contained in *Trifolium repens* and mixture nitrogen concentration / isotope composition.

Relationship between the proportion of total nitrogen contained in *T. repens* and (a) nitrogen concentration and (b) nitrogen isotopic composition of a *T.-repens-Lolium-perenne* mixture.
